# Response Time Dynamics From Noncognitive Ordinal Ecological Momentary Assessment as a Proxy for Symptom Change in Geriatric Depression: Longitudinal Observational Study

**DOI:** 10.2196/83891

**Published:** 2026-05-08

**Authors:** Jooho Lee, Jeehang Lee, Sehwan Park, Gangho Do, Jihye Noh, Sangjoon Moon, Kyungmi Chung, Sang Joon Son, Jin Young Park

**Affiliations:** 1 Medical Research Team, Digital Medic Co., Ltd., Seoul, Republic of Korea; 2 Department of Human-Centered Artificial Intelligence, College of Intelligence Information Engineering, Sangmyung University, Seoul, Republic of Korea; 3 Center for Digital Health, Yongin Severance Hospital, Yonsei University Health System, Yongin, Republic of Korea; 4 Department of Psychiatry, Yongin Severance Hospital, Yonsei University College of Medicine, 363, Dongbaekjukjeon-daero, Giheung-gu, Yongin, 16995, Republic of Korea, 82 31-5189-8148, 82 31-5189-8565; 5 Institute of Behavioral Science in Medicine, Yonsei University College of Medicine, Yongin, Republic of Korea; 6 Department of Psychiatry, Ajou University School of Medicine, Suwon, Republic of Korea

**Keywords:** ecological momentary assessment, response time, digital biomarkers, geriatric depression, Bayesian multilevel modeling, self-monitoring, mobile health

## Abstract

**Background:**

Depressive symptoms in older adults are amplified by social isolation and limited access to clinic-based mental health care. Ecological momentary assessment (EMA) enables remote self-monitoring and unobtrusively captures response times (RTs), which may serve as indicators of psychomotor and cognitive functioning.

**Objective:**

This study investigated the use of EMA-based RT dynamics for predicting symptom change and profiling potential responders for repeated self-monitoring in late-life depression.

**Methods:**

Forty-nine community-dwelling adults aged 65 years or older (mean age 70.7, SD 5.8 years; female: 35; male: 14) with a history of major depressive disorder received case management incorporating daily EMA. Participants provided self-reports of mood, appetite, sleep quality, and general well-being. Preassessment and postassessment included the 15-item Short Geriatric Depression Scale (GDS-15), the Center for Epidemiologic Studies Depression Scale-Revised (CESD-R), the 9-item Patient Health Questionnaire, and the Beck Anxiety Inventory. RTs were cleaned with an asymmetric IQR rule, *z* standardized within-person × response level, and modeled with exponential decay curves over successive EMA trials. The efficacy of EMA-adjunctive care was evaluated using pre-post comparisons of symptom scales. We then examined associations between RT-derived features and symptom change using correlational analyses. Finally, Bayesian multilevel modeling was applied to assess the clinical relevance of RT dynamics, including group differences in adaptation patterns.

**Results:**

Older adults at risk for depression showed significant symptom reductions over the 4-week EMA-adjunctive care period across all 4 psychological scales (CESD-R: mean Δ 11.5; rank-biserial *r*=0.78; GDS-15: mean Δ 2.14, Cohen *d*=0.76), alongside high EMA adherence (>90%). In correlational analyses, descriptive EMA score metrics and raw RTs showed modest, symptom-specific associations with symptom change (ΔCESD-R: |*r*|≈0.29; Δ9-item Patient Health Questionnaire: |*r*|≈0.32; ΔBeck Anxiety Inventory: |*r*|≈0.35) but were not significantly related to change in geriatric depression (ΔGDS-15: |*r*|≈0.24). In contrast, exponential-decay model parameters derived from standardized RT were significantly associated with geriatric depressive symptom change (Δ GDS-15), with the strongest effects observed for the feeling item (eg, decay rate *θ*_*b*_: *r*=−0.398, asymptote *θ*_*c*_: *r*=−0.321). Bayesian multilevel modeling further indicated that EMA-adjunctive care responders showed faster RT adaptation than nonresponders (median decay-rate ratio≈4.9, 95% credible interval 1.44-14.31), whereas differences in postadaptation RT levels were smaller and uncertain (median postadaptation RT ratio≈1.25, 95% credible interval 0.95-1.58). Sensitivity analyses showed consistent decay-rate effects across alternative specifications.

**Conclusions:**

Dynamic characteristics of EMA-based RTs emerged as a sensitive proxy for monitoring changes in depressive symptoms among older adults at risk. These findings highlight the potential use of RTs as digital biomarkers derived from brief, low-burden EMA self-monitoring, supporting the development of scalable and personalized mental health interventions for geriatric populations.

## Introduction

### Modern Hazards of Late-Life Depression

Population aging has brought renewed attention to late-life depression. Growth in single-person households, shrinking family networks, and weaker community ties can intensify the risk of depression in older adults, particularly in highly urbanized settings, such as South Korea [1,2]. These shifts also strain clinic-centered care models, which are often inaccessible to older adults living alone and facing mobility barriers, underscoring the need for remote, daily-life–responsive geriatric mental health strategies.

### Ecological Momentary Assessment: From Monitoring to Care

Ecological momentary assessment (EMA) involves the real-time, in situ measurement of behaviors, emotions, and thoughts in everyday contexts [3,4]. By capturing experiences as they occur, EMA reduces recall bias and is well suited to symptoms that fluctuate with context [4]. Advances in mobile and sensor technologies have enabled unobtrusive, scalable EMA deployment, positioning it as a potential infrastructure for practical routine mental health support methods [5,6]. Repeated self-monitoring via EMA may also confer therapeutic benefits, particularly when combined with standard treatments [7-11]; however, effects appear heterogeneous, with reduced responsiveness among individuals with more severe baseline symptoms, highlighting the need to identify who benefits most from EMA-based approaches [12].

### EMA Response Times: Linking Cognition and Depression in Older Adults

Recent EMA research has moved beyond self-reported content to “side data” such as response time (RT), which may index underlying cognitive processes. Chung et al [13] reported a nonlinear (inverted U-shaped) association between EMA RT and depression severity, suggesting that both unusually fast and slow responses may reflect dysfunction (eg, impulsivity or cognitive slowing). Hernandez [14] further showed that EMA RTs correlate with Symbol Search performance, indicating that RTs capture general processing capacity even in noncognitive EMA tasks.

EMA-derived RTs may therefore help identify individuals vulnerable to depressive symptoms. Consistent with *Diagnostic and Statistical Manual of Mental Disorders, Fifth Edition* (*DSM-5*) descriptions of major depressive disorder, which include observable psychomotor retardation [15], Hernandez et al [16] applied drift diffusion modeling to binarized EMA responses and found that RT-derived parameters (eg, drift rate and boundary separation) systematically tracked traits such as neuroticism and depressive symptoms [16]. Together, these findings position EMA RT as a candidate marker linking cognitive efficiency with affective symptomatology.

This approach may be particularly relevant in geriatric populations, where aging and depression jointly exacerbate psychomotor slowing [17-19]. Older adults with major depressive disorder (MDD) show prolonged initiation and movement times relative to controls, implicating combined executive and motor delays [17], and reviews report especially large effects for psychomotor slowing in late-life samples [20]. Depressed older adults also appear less adaptable under repeated cognitive demands: greater symptom severity has been associated with slower reaction-time learning curves and reduced improvement across training blocks, with sluggish adaptation predicting later functional decline [21,22]. Some interventions may even backfire when effort exceeds coping capacity; for instance, one randomized controlled trial reported worsening depressive symptoms following speed-of-processing training [23]. Collectively, these results motivate EMA-based RT metrics as scalable markers for monitoring cognitive-motor slowing and adaptation in late-life depression.

### Challenges in Utilizing Ordinal-Scale, Noncognitive EMA RTs

Despite its potential usage, analyzing RTs from ordinal-scale EMA items presents distinct hurdles. While ordinal items, such as 5- or 7-point Likert scales, offer richer granularity than binary formats, their integration with RT data poses additional analytical considerations and requires further preprocessing steps.

One complication arises from emotional clarity. As shown in Hernandez et al [16], individuals with clearer emotional awareness due to intense emotions tend to respond more quickly to EMA questionnaires. Thus, trait-level factors, such as baseline emotional reactivity, as well as situational factors, including bereavement, acute illness, and exposure to daily stressors, may affect the RT of EMA reports. This can mask or confound the expected slowing associated with cognitive impairment, making RT a less straightforward marker when emotional clarity varies across individuals and between occasions.

A further complication lies in interface variability. Mobile EMA apps often use different visual formats (eg, sliders, buttons, or scales), which can unintentionally influence how quickly users respond. For example, a response option that is most proximal to a frequently used finger can lead to faster responses. These app-specific differences, such as user interface composition, introduce another layer of noise that must also be accounted for when analyzing ordinal-scaled EMA-based RT.

### Research Purpose

In light of these issues, this study pursues the following objectives. First, we tested whether short-term EMA use (≤4 wk) alongside usual case management is associated with symptom improvement among older adults at elevated risk for depression, evaluating EMA as a potential adjunctive intervention rather than a monitoring-only tool. Second, we examined how multiple EMA-derived features (eg, symptom ratings, response speed, and RT adaptation) relate to symptom change over time, with a focus on RT dynamics as a behavioral signature of improvement. We also introduce a scalable analytic approach that reduces key ecological confounds in RT (eg, momentary affect intensity and interface-related heterogeneity) and is broadly applicable across EMA apps and Likert-type scales. Third, we evaluated the clinical relevance of RT adaptation by using a Bayesian multilevel model to compare adaptive patterns between participants who benefited from EMA-adjunctive care and those who did not. With a modest sample size (n=50), partial pooling in a multilevel framework improves estimate stability while accommodating missing and unbalanced EMA observations [24]. Additionally, Bayesian posterior inference provides interpretable uncertainty for group differences in adaptation parameters, thereby quantifying the evidence for between-group differences in adaptive patterns [24-26]. This research ultimately supports the objective, remote stratification of individuals who are most likely to benefit from EMA-enabled care.

## Methods

### Eligibility Criteria

Participants eligible for this study were aged 65 years or older, regardless of gender, and had expressed their willingness to participate through recruitment notices posted at the community-based Suwon Geriatric Mental Health and Welfare Center in Gyeonggi-do, Republic of Korea. Individuals who met the following eligibility criteria were included: (1) participants must have had a prior diagnosis of depressive disorder by a psychiatrist, with no change in medication for the last 3 months and without symptom aggravation; (2) absence of medical or neurological disorders such as dementia, stroke, or Parkinson disease that may affect the research; and (3) current recipients of case management services at the Suwon Geriatric Mental Health and Welfare Center.

### Ethical Considerations

All participants who met these criteria provided written informed consent prior to enrollment and completed demographic information and preassessment questionnaires. Those who completed these surveys were then given detailed guidance and support for installing and using the app, with additional assistance provided to those less familiar with digital tools. Given the specific population’s limited experience with digital devices, only individuals who demonstrated the ability to follow the mobile app (BIG4+) usage guidelines independently were enrolled. Participation was entirely voluntary, and participants were free to withdraw at any time without any disadvantage. All procedures were approved by the Public Institutional Bioethics Committee designated by the Ministry of Health and Welfare of the Korean Government (MOHW; P01-202405-01-019) and were supported by Digital Medic Inc. Written informed consent was obtained from all participants. Participant privacy was protected by analyzing all data in deidentified form and restricting access to authorized research staff only. Electronic data were stored in password-protected encrypted files, and physical records were kept in locked storage. Participants received KRW 100,000 (approximately US $67.50) for completing the full study protocol. For early withdrawal, compensation was provided on a prorated basis according to the week of completion.

### Research Design

The study used an observational, single-arm design. Participants were community-dwelling older adults enrolled through the Suwon Geriatric Mental Health and Welfare Center.

Case management was provided throughout the study, consisting of 1 monthly home visit and 1 to 2 interim telephone contacts, during which case managers monitored participants’ daily living conditions and mental health status. In addition to this standard case management, participants were asked to use the Big4+ mobile app as an adjunctive care tool over a 4-week period. The Big4+ app, developed specifically for research purposes, was available on both Android (AOS) and iOS platforms and collected daily self-reports on mood, appetite, sleep quality, and overall well-being using a 7-point Likert scale (1=minimum to 7=maximum).

Each participant visited the center twice: once prior to initiating app usage (baseline assessment, V1) and once at the end of the 4-week period (postassessment, V2). During both visits, participants completed a battery of self-report questionnaires administered in paper format by case managers, with each session lasting approximately 30 minutes.

Standardized psychological scales administered to participants included the Korean version of the Center for Epidemiologic Studies Depression Scale-Revised (CESD-R) [27-29], the Korean version of the 15-item Short Geriatric Depression Scale (GDS-15) [30,31], the 9-item Patient Health Questionnaire-9 (PHQ-9) [32,33], and the Beck Anxiety Inventory (BAI) [34,35].

### Data Preprocessing

#### Handling RT Outliers

To the best of our knowledge, there is no established gold standard for identifying outliers in RTs associated with noncognitive and ordinal EMA items. Furthermore, the population of this study (older adults at high risk for depression) was highly specific, making it necessary to define acceptable RTs empirically based on the distribution within the dataset. We adopted the IQR rule, where the IQR is calculated as the difference between the third quartile and the first quartile. Outlier boundaries were defined as specific multiples of the IQR from Q1 and Q3. Given that RT distributions were heavily right-skewed, we applied asymmetric boundaries across the dataset: a lower bound of Q1 – 0.5 × IQR and an upper bound of Q3 + 1.5 × IQR. These boundaries were calculated separately for each EMA item (feeling, appetite, feeling of sleep, and general evaluation). Trials with RTs falling outside these bounds were excluded from the analysis. The details of the predefined outlier thresholds and the decision-making process are provided in Section I in Multimedia Appendix 1.

#### Standardization of RTs

EMA RTs are noisy and can be confounded by momentary affect (eg, faster responses during more intense or clearly defined states) and device or app characteristics [16]. To improve comparability, we standardized RTs within individuals and response options, expressing each latency relative to that participant’s typical speed at a given Likert level.

For individual *i*, response option *j* (1-7), and trial *k*, standardized RT (Z-RT) was as follows:


$$Z_{ij}^{\left(k\right)}=\frac{RT_{ij}^{\left(k\right)}-{\overset{‾}{RT}}_{ij}}{\sigma_{ij}},$$

where RT_*ij*_ and *σ*_*ij*_ are the mean and SD of RT for participant *i* at option *j*. This z-transformation reduces between-context and between-person variability, supporting more reliable downstream analyses.

#### Extracting EMA-Based Digital Features

Descriptive features were extracted from 4 weeks of EMA data collected from each participant. For each EMA item, we computed the mean, SD, minimum, maximum, and interquartile values (25th, 50th, and 75th percentiles) of the reported scores to characterize overall mood patterns throughout the study period. In addition, identical descriptive statistics of EMA-based RTs were derived as indicators of cognitive processing speed and consistency.

Beyond overall response latency, we quantified how participants adapted to repeated EMA self-reflection over time. Such “practice effects” (ie, progressive reductions in RT with repeated exposure) are widely documented in cognitive tasks and are relevant to EMA given evidence that even noncognitive RTs reflect cognitive traits [16]. Although practice effects are often modeled with power-law decay [36], exponential decay may better capture adaptation in older adults, who typically show slower motor and cognitive adjustment [37,38]. Accordingly, we fit an exponential decay curve to each participant’s longitudinal RT series (both raw RT and standardized RT):


$$f\left(t\right)=\theta_{a}\cdot exp\left(-\theta_{b}\cdot t\right)+\theta_{c},$$

where *t* indexes time or trials. Parameters were estimated via nonlinear least squares (Levenberg-Marquardt; scipy.optimize.curve_fit; max 10,000 iterations). The parameter *θ_a_* (amplitude) captures the initial elevation in RT above the asymptote (ie, *f* (0)–*θ*_*c*_ ), reflecting the potential magnitude of improvement across repeated trials. The parameter *θ_b_* (decay rate) indexes the speed of adaptation, indicating how rapidly an individual becomes accustomed to repeated self-monitoring. The parameter *θ*_*c*_ (asymptote or postadaptation RT) represents the asymptotic minimum RT, interpreted as the stabilized latency after maximal adaptation within the observation window. The model was fit to both raw and standardized RTs, with parameter bounds set to match the scale and range of each outcome.

In this stage, exponential decay parameters were estimated separately for each participant (ie, without partial pooling) to serve as person-level features that can be computed in naturalistic settings where no external reference data are available. This individual-fit approach also facilitates a fair comparison with other EMA-derived features (eg, self-report scores and RT descriptives), which were likewise calculated at the individual level without borrowing strength from group-level information. Technical details regarding the fitting of the exponential-decay model are provided in Section II in Multimedia Appendix 1.

#### Defining Symptom Improvement

A key step prior to the analyses was to select an appropriate metric for symptom change. Although treatment response is often operationalized using simple change scores (post-pre), this approach has been criticized for its dependence on baseline severity and the resulting negative correlation with baseline values [39-41]. Percentage change scores adjust for baseline differences and are widely used in other clinical domains [42,43] but can be difficult to interpret and may reduce statistical power due to skewed distributions [40,41]. Regression-based alternatives (eg, analysis of covariance or residualized change) can control for baseline levels yet may yield biased estimates when baseline severity is related to treatment effects, which is common in observational settings [40,41]. Given these considerations and prior methodological work supporting the robustness of simple change scores in nonrandomized contexts [40,44,45], we used simple change score Δ(post-pre) as the primary symptom improvement outcome.

#### Responder Classification (Minimally Detectable Change Criteria)

Participants were classified as “responders” or “nonresponders” to EMA-adjunctive care based on minimally detectable change (MDC) thresholds derived from the GDS-15. A recent meta-analysis by González-Domínguez et al [46], which included 5876 older adults across 21 studies (mean age 76 y), reported that a score reduction of 3.81 points on the GDS-15 corresponds to the MDC threshold, defined as the level of change with only a 5% probability of being attributable to measurement error (95% CI 3.59-4.04) [47]. Based on these findings, an MDC threshold of –3.81 points was applied, reducing the arbitrariness of classification and accounting for measurement error.

### Analysis

#### Explorative Effects of EMA-Based Self-Monitoring

Explorative analysis was conducted to assess the benefits of EMA-adjunctive care in older populations. Given the single-arm design, the effectiveness of EMA-based self-monitoring in geriatric depression was assessed by comparing symptom scores collected at baseline (V1) and postintervention (V2) using paired statistical tests. The choice of test was determined based on the normality of the difference scores, as assessed by the Shapiro-Wilk test. When the assumption of normality was satisfied, a paired-samples *t* test was applied. In cases where the normality assumption was violated, the nonparametric Wilcoxon signed-rank test was used instead. All paired tests in this analysis were 1-tailed.

#### Association Between EMA-Derived Features and Symptom Improvement

We examined whether EMA-derived features, particularly RT dynamics, track symptom improvement using correlational analyses. We computed Spearman correlations between EMA-derived features and Δ on the GDS-15 and assessed specificity using parallel analyses for the CESD-R, PHQ-9, and BAI.

#### Clinical Relevance of EMA Trajectories for Symptom Improvement

To examine the clinical relevance of EMA-based RT dynamics, we fitted a moderated Bayesian multilevel exponential-decay model to test whether adaptive patterns of EMA-based RTs differed by the MDC group. The model equation is demonstrated as a fully randomized model. For detailed model specifications and rationales, see Section III in Multimedia Appendix 1.

##### Observation Model

For each observation n=1, ..., N_obs_,


$$RT_{n}∼\text{LogNormal}\left(\eta_{n},\sigma_{log}\right)\Leftrightarrow log\left(RT_{n}\right)∼N\left(\eta_{n},\sigma_{log}^{2}\right),$$

we assumed each observation follows a log-normal distribution, ensuring positive predictions and accommodating the right-skew typical in RT data.

##### Mean Structure (RT Adaptation)

Expected log-RT followed a subject-specific exponential decay over normalized time *t*∈ [0, 1]:


$$\eta_{n}=\theta_{a}^{\left(i\right)}\cdot \exp\left(-\theta_{b}^{\left(i\right)}\cdot t\right)+\theta_{c}^{\left(i,r\right)},$$

where *i* indexes subjects and *r*∈ 1, ..., *R* indexes response options (Likert-7 scale).

##### Multilevel Effects and MDC Moderation

Amplitude and decay rate were modeled on the log scale (log-linked) for positive constraints and allowed to vary by subject, with MDC group moderation:


$$\begin{array}{rl} \log\theta_{a}^{\left(i\right)} & =\beta_{0a}+\beta_{1a}\cdot g^{\left(i\right)}+\sigma_{a}\cdot z_{a}^{\left(i\right)},\;z_{a}^{\left(i\right)}∼N(0,1),\\ \log\theta_{b}^{\left(i\right)} & =\beta_{0b}+\beta_{1b}\cdot g^{\left(i\right)}+\sigma_{b}\cdot z_{b}^{\left(i\right)},\;z_{b}^{\left(i\right)}∼N(0,1). \end{array}$$

Baseline log-RT or postadaptation RT (𝜃_𝑐_^(𝑖,𝑟)^) was decomposed into group, subject, and response-option components:


$$\theta_{c}^{\left(i,r\right)}=\underset{\text{Group-Moderated Baseline}}{\underbrace{\left(\beta 0_{c}+\beta 1_{c}\cdot g^{\left(i\right)}\right)}}+\underset{\text{Subject Intercept}}{\underbrace{u_{c}^{\left(i\right)}}}+\underset{\text{Option Main Effect}}{\underbrace{\alpha_{c}^{\left(r\right)}}}+\underset{\text{Cell Residual}}{\underbrace{\epsilon_{c}^{\left(i,r\right)}}}$$

with $u_{c}^{\left(i\right)}∼N\left(0,\sigma_{\text{subj}}^{2}\right)$, $\epsilon_{c}^{\left(i,r\right)}∼N\left(0,\sigma_{\text{cell}}^{2}\right)$, and $\sum_{r=1}^{R}\alpha_{c}^{\left(r\right)}=0$ for identifiability. The decomposition of baseline log-RT or postadaptation RT aligns with the framework advocating for the within-person and within-response option standardization of RTs. By explicitly modeling these components, the model accounts for response-option biases in observed latency while simultaneously partitioning subject-specific baseline variance.

##### Priors and Inference

We used weakly informative priors $\beta 0_{*}∼N\left(\mu_{\text{emp}},3^{2}\right)$, $\beta 1_{*}∼N\left(0,3^{2}\right)$, $\alpha_{c}^{\left(r\right)}∼N\left(0,1^{2}\right)$, *σ*_*a*_, *σ*_*b*_, *σ*_subj_, *σ*_cell_∼HalfNormal(1.5), and *σ*_log_∼HalfNormal(1). Models were fit in PyMC using NUTS (4 chains; 1000 warm-up + 1000 draws per chain; target acceptance = 0.95) [48].

To determine the appropriate degree of hierarchical structure in EMA-based RT trajectories, we compared a set of nested Bayesian exponential-decay models that differed only in their random-effects specification. Specifically, we evaluated (1) a nonhierarchical model with fixed parameters across participants, (2) partially hierarchical variants allowing subject-level variation in selected parameters (eg, random *θ*_*b*_ only; random *θ*_*a*_ and *θ*_*b*_; random *θ*_*b*_ and $\theta_{c}^{\left(i,r\right)}$), and (3) a full hierarchical model with subject-level variation in key components. Models were compared using Pareto-smoothed importance sampling leave-one-out cross-validation (PSIS-LOO), summarizing out-of-sample predictive performance via the expected log pointwise predictive density leave-one-out cross-validation (ELPD-LOO). To isolate the contribution of random effects, MDC-group moderation terms were omitted during this model-structure comparison. Based on the best-performing random-effects structure (selected model; random *θ*_*b*_ and *θ*_*c*_, with *θ*_*a*_ fixed across subjects), we then tested clinical relevance by extending the selected model to include MDC group moderation, allowing these parameters to vary systematically between responders and nonresponders.

Group differences were quantified using Bayesian posterior summaries rather than frequentist hypothesis tests. For log-linked parameters (eg, *θ*_*a*_, *θ*_*b*_), effects were expressed as responder to nonresponder multiplicative ratios exp (*β*1); for baseline parameters on the log-RT scale (eg, *θ*_*c*_), group effects were additive on the log scale and additionally converted to RT ratios via exponentiation. We report the posterior median of the responder-to-nonresponder ratio, computed draw-by-draw from the posterior samples, along with the 95% credible interval (CrI) for this ratio. A ratio of 1 indicates no difference between the groups on the original scale; ratios greater than 1 indicate larger parameter values in responders (eg, a ratio of 1.30 corresponds to ~30% higher values), whereas ratios less than 1 indicate smaller values in responders relative to nonresponders. The 95% CrI represents the range containing 95% of the posterior probability for the quantity (given the specified model and priors). We also report the posterior probability of an increase, *P*(Ratio_Responder/Nonresponder_ > 1|data), which quantifies the evidence that responders have larger parameter values than nonresponders.

Models were fit separately for each EMA item and additionally for an averaged RT outcome across items; for the averaged outcome, continuous scores were discretized into 7 bins to align with the response-option structure used in item-level models. Finally, we conducted sensitivity analyses by refitting the model under alternative weakly informative priors (Student *t*) and a more complex plausible random-effects specification and verified that key group effect conclusions were robust across these reasonable modeling choices.

## Results

### Participants

A total of 50 older adults aged 65 years or older were recruited for the study (mean age 70.6, SD 5.8 y), of whom 72% (n=35) were female. Among the participants, 49 had a documented history of MDD, and 1 had a history of bipolar disorder. To ensure consistency and avoid confounding effects arising from the natural mood fluctuations associated with bipolar disorder, only participants with MDD were included in all statistical analyses (n=49). Baseline psychological assessments indicated clinically elevated symptoms across multiple domains. Preassessment scores were as follows: GDS-15 mean=9.37 (SD 4.42), CESD-R mean=33.94 (SD 18.24), PHQ-9 mean=13.04 (SD 7.40), and BAI mean=22.76 (SD 16.46). Following the study period, scores were as follows: GDS-15 mean=7.22 (SD 4.44), CESD-R mean=22.65 (SD 16.15), PHQ-9 mean=8.59 (SD 6.30), and BAI mean=13.31 (SD 11.52). All participants successfully installed and used the BIG4+ app over the 4-week period, with a mean compliance rate above 93%. All descriptive details are summarized in Table 1.

**Table 1.** Descriptives of demographics, psychological assessments, and mobile app (BIG4+) compliance (N=49).

Descriptives	Values
Gender, n (%)	
Women	35 (71.4)
Men	14 (28.6)
Age (y), mean (SD)	70.7 (5.8)
Operating system, n (%)	
Android	49 (100)
iOS	0 (0)
Income level, n (%)	
Lowest	0 (0)
Low	6 (12.2)
Middle	15 (30.6)
High	8 (16.3)
Highest	18 (36.7)
No response	2 (4.1)
Residence type, n (%)	
Living with family	20 (40.8)
Living alone	29 (59.2)
Relationship with family, n (%)	
Very bad	5 (10.2)
Bad	8 (16.3)
Moderate	16 (32.7)
Good	15 (30.6)
Very good	4 (8.2)
No response	1 (2.0)
Intimate person present, n (%)	
Yes	28 (57.1)
No	19 (38.8)
No response	2 (4.1)
Belonging group present, n (%)	
None	15 (30.6)
1 group	19 (38.8)
2 groups	8 (16.3)
3 groups	5 (10.2)
Above 4 groups	2 (4.1)
Preassessment scale (V1), mean (SD)	
GDS-15^a^	9.4 (4.4)
CESD-R^b^	33.9 (18.2)
PHQ-9^c^	13 (7.4)
BAI^d^	22.8 (16.5)
Postassessment scale (V2), mean (SD)	
GDS-15	7.2 (4.4)
CESD-R	22.7 (16.1)
PHQ-9	8.6 (6.3)
BAI	13.3 (11.5)
BIG4+ adherence (%), mean (SD)	93.8 (9.1)

a GDS: 15-item Geriatric Depression Scale.

b CESD-R: Center for Epidemiologic Studies Depression Scale-Revised.

c PHQ-9: 9-item Patient Health Questionnaire.

d BAI: Beck Anxiety Inventory.

### EMA-Adjunctive Care and Short-Term Symptom Improvement in at-Risk Older Adults

To evaluate the benefit of EMA-based self-monitoring, preassessment and postassessment scores were compared across 4 psychological scales (GDS-15, CESD-R, PHQ-9, BAI). The results indicated that CESD-R, PHQ-9, and BAI scores violated the assumption of normality (*P* values =.01, .01, and .01, respectively), while the GDS-15 difference scores did not (*P*=.33). Consequently, Wilcoxon signed-rank tests were applied to the CESD-R, PHQ-9, and BAI, and a paired-samples *t* test was used for the GDS-15 (Table 2).

All 4 measures showed statistically significant improvements following the intervention. CESD-R scores decreased by a mean of 11.50 points (SD 15.8; SE 2.26; *W*=1045.00; *P*<.001), with a rank biserial correlation of 0.78. PHQ-9 scores decreased by a mean of 4.50 points (SD 6.9; SE 1.00; *W*=947.00; *P*<.001), with an effect size of 0.75. BAI scores were reduced by 9.00 points (SD 11.7; SE 1.67; *W*=940.00; *P*<.001), with a rank biserial correlation of 0.82. GDS-15 scores showed a significant reduction of 2.14 points (SD 2.8; SE 0.40; *t*_48_= 5.30; *P*<.001), corresponding to a Cohen *d* of 0.76.

**Table 2.** Paired tests of psychological assessment scores between preintervention (V1) and postintervention (V2).

Scales	Test	Statistics	*P* value	Rank biserial correlation	Cohen *d*
GDS-15^a^	Student *t*	5.30	<.001	—^b^	0.76
CESD-R^c^	Wilcoxon *W*	1045.0	<.001	0.78	—
PHQ-9^d^	Wilcoxon *W*	947.0	<.001	0.75	—
BAI^e^	Wilcoxon *W*	940.0	<.001	0.82	—

a GDS-15: 15-item Geriatric Depression Scale.

b Not applicable.

c CESD-R: Center for Epidemiologic Studies Depression Scale-Revised.

d PHQ-9: 9-item Patient Health Questionnaire.

e BAI: Beck Anxiety Inventory.

### Associations Between EMA-Derived Features and Symptom Improvement

Linear relationships between EMA-derived features and changes in depressive and anxiety symptoms were examined using correlational analyses. A full table of correlational analyses is presented in Multimedia Appendix 2.

Descriptive statistics of EMA scores showed generally small-to-modest associations with mental health change scores (Table 3), with no significant correlations for ΔGDS-15 across items. Significant associations emerged for other outcomes, suggesting item-specific links with symptom change: ΔCESD-R was correlated with the minimum of appetite (*r*=0.294; *P*=.04) and general evaluation (*r*=0.29; *P*=.04). ΔPHQ-9 was correlated with the median of feeling (*r*=0.291; *P*=.04), mean (*r*=0.325; *P*=.02), and minimum of appetite rating (*r*=0.291; *P*=.04), and the minimum of general evaluation (*r*=0.286; *P*=.046). For anxiety, ΔBAI was associated only with the minimum sleep quality rating (*r*=0.335; *P*=.02).

**Table 3.** Associations between mental health change scores (Δ), EMA score, and EMA response time descriptives.^a^

Type, EMA^b^ item, and feature	ΔGDS-15^c^ (*r^p^*)	ΔCESD-R^d^ (*r^p^*)	ΔPHQ-9^e^ (*r^p^*)	ΔBAI^f^ (*r^p^*)
EMA score				
Feeling				
Mean	0.094	0.239	0.251	0.204
SD	0.074	–0.246	–0.193	–0.255
Minimum	–0.01	0.267	0.234	0.26
Median	0.039	0.252	0.291*	0.234
Maximum	0.233	0.001	0.062	0.025
Appetite				
Mean	0.241	0.273	0.325*	0.152
SD	0.145	–0.221	–0.136	–0.163
Minimum	0.013	0.294*	0.291*	0.14
Median	0.193	0.245	0.264	0.107
Maximum	0.263	0.006	0.086	–0.07
Sleep quality				
Mean	0.119	0.074	0.197	0.16
SD	–0.004	–0.231	–0.123	–0.192
Minimum	–0.06	0.245	0.277	0.335*
Median	0.116	0.06	0.141	0.085
Maximum	0.028	0.00	0.038	0.042
General evaluation				
Mean	0.121	0.168	0.198	0.149
SD	0.016	–0.26	–0.196	–0.203
Minimum	0.029	0.29*	0.286*	0.261
Median	0.106	0.156	0.175	0.162
Maximum	0.024	0.072	0.04	0.101
EMA response time				
Feeling				
Mean	0.091	–0.073	0.2	–0.108
SD	0.092	0.074	0.091	–0.146
Minimum	0.099	–0.028	0.17	–0.039
Median	0.101	–0.129	0.175	–0.164
Maximum	0.107	0.103	0.181	–0.05
Appetite				
Mean	0.195	–0.122	0.037	–0.301*
SD	0.008	–0.141	–0.077	–0.282
Minimum	0.105	–0.114	0.04	–0.158
Median	0.229	–0.053	0.077	–0.261
Maximum	0.044	–0.159	–0.049	–0.35*
Sleep quality				
Mean	0.251	–0.067	0.011	–0.224
SD	0.189	–0.003	–0.106	–0.023
Minimum	0.17	–0.104	0.053	–0.139
Median	0.257	–0.015	0.078	–0.189
Maximum	0.143	–0.079	–0.13	–0.13
General evaluation				
Mean	0.084	–0.071	0.129	–0.034
SD	0.201	–0.148	–0.13	–0.159
Minimum	–0.004	–0.09	0.097	–0.018
Median	0.083	–0.024	0.146	–0.034
Maximum	0.138	–0.193	–0.068	–0.182

a Significance thresholds: *P<.05, **P<.01, ***P<.001.

b EMA: ecological momentary assessment.

c GDS-15: 15-item Geriatric Depression Scale.

d CESD-R: Center for Epidemiologic Studies Depression Scale-Revised.

e PHQ-9: 9-item Patient Health Questionnaire.

f BAI: Beck Anxiety Inventory.

Descriptive statistics of raw EMA RTs showed limited associations with symptom change (Table 3). No RT features were significantly correlated with ΔGDS-15, ΔCESD-R, or ΔPHQ-9. The only significant RT associations were observed for anxiety change (ΔBAI), where mean (*r*=–0.301; *P*=.04) and maximum (*r*=–0.35; *P*=.01) of appetite RT were negatively correlated with ΔBAI, underscoring the challenge of using noisy, naturalistic raw RT descriptives to track depressive symptom change in at-risk older adults.

In contrast, exponential-decay parameters derived from Z-RT (within subject×response-option standardization) showed clearer associative patterns (Table 4). For the feeling item, both the decay rate and asymptote were significantly associated with ΔGDS-15 (*θ*_*b*_: *r*=–0.398; *P*=.005, *θ*_*c*_: *r*=–0.321; *P*=.03), representing the strongest ΔGDS-15 correlations among the RT-derived features and exceeding those observed for EMA score descriptives. Feeling-item parameters also showed significant associations with other outcomes: ΔCESD-R was related to model fit and dynamics (*R*^2^: *r*=0.358; *P*=.01, *θ*_*b*_: *r*=–0.302; *P*=.04, *θ*_*c*_: *r*=–0.305; *P*=.04), while ΔBAI was associated only with goodness-of-fit (*R*^2^: *r*=0.33; *P*=.02). Notably, no significant associations were observed for decay parameters fitted to appetite, sleep quality, or general evaluation across change scores (all P>.05), and no Z-RT decay features were significantly related to ΔPHQ-9.

**Table 4.** Associations between mental health change scores and EMA Z-RT exponential decay parameters.^a^

EMA^b^ item and RT^c^ parameter		ΔGDS-15^d^ (*r*^*p*^)	ΔCESD-R^e^ (*r*^*p*^)	ΔPHQ-9^f^ (*r*^*p*^)	ΔBAI^g^ (*r*^*p*^)
Feeling					
*R*^2^		0.19	0.358*	0.271	0.330*
*θ*_*a*_		0.209	0.144	0.017	0.244
*θ*_*b*_		−0.398**	−0.302*	−0.263	−0.186
*θ*_*c*_		−0.321*	−0.305*	−0.266	−0.166
Appetite					
*R*^2^		0.034	0.198	0.206	0.266
*θ*_*a*_		0.047	0.17	0.158	0.188
*θ*_*b*_		0.23	0.072	−0.038	−0.12
*θ*_*c*_		0.172	0.004	−0.084	−0.153
Sleep quality					
*R*^2^		−0.194	−0.196	−0.28	−0.021
*θ*_*a*_		0.107	0.165	0.042	0.206
*θ*_*b*_		0.182	0.137	0.213	−0.059
*θ*_*c*_		0.099	0.145	0.229	0.041
General evaluation					
*R*^2^		0.206	−0.045	−0.05	−0.15
*θ*_*a*_		0.242	0.032	−0.042	−0.054
*θ*_*b*_		−0.104	0.227	0.181	0.127
*θ*_*c*_		−0.057	0.119	0.155	0.213

a Significance thresholds: *P<.05, **P<.01, ***P<.001.

b EMA: ecological momentary assessment.

c RT: response time.

d GDS-15: 15-item Geriatric Depression Scale.

e CESD-R: Center for Epidemiologic Studies Depression Scale-Revised.

f PHQ-9: 9-item Patient Health Questionnaire.

g BAI: Beck Anxiety Inventory.

### Group Differences in RT Trajectories by EMA-Adjunctive Care Response

#### Bayesian Multilevel Modeling

We next evaluated the clinical relevance of EMA-based RT trajectories in relation to depressive symptom improvement during EMA-adjunctive care. Responder-status moderation was tested via the posterior distribution of the group term (*β*1) in a Bayesian multilevel exponential-decay model, where responders were defined as participants whose GDS-15 change exceeded the MDC threshold (ΔGDS-15≤–3.81). Because prior correlational analyses indicated that only RT dynamic parameters from the feeling item were reliably associated with ΔGDS-15, the following results focus on models fit to feeling-item RT (including model selection, adequacy checks, and group effects). Group differences are summarized using posterior estimates, responder-to-nonresponder parameter ratios, and posterior probabilities of increase.

#### Model Selection (Random-Effects Structure)

**Figure 1.**
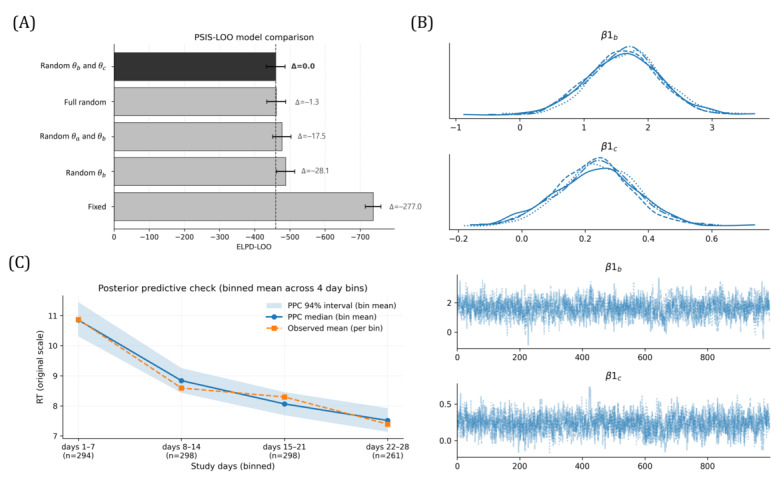
Model selection and diagnostic summary. (A) Pareto-smoothed importance sampling leave-one-out (PSIS-LOO) expected log predictive density (ELPD) by random-effects structure (higher is better). (B) Markov chain Monte Carlo (MCMC) trace plots for responder-status moderation terms (*β*1) on key parameters (*θ*_*b*_ and *θ*_*c*_). (C) Binned posterior predictive check comparing observed response time (RT) summaries with posterior predictive medians and 94% intervals. PPC: posterior predictive check.

PSIS-LOO model comparison favored a partial hierarchical exponential-decay model with subject-level random effects on the decay rate (*θ*_*b*_) and postadaptation RT (*θ*_*c*_) (Figure 1A; ELPD-LOO=−459.7). A full random-effects model performed similarly (ΔELPD-LOO=1.3) but showed higher effective complexity ($p_{\text{loo}}$: 112.2 >109.5), indicating limited predictive benefit from additionally allowing subject-specific variability in amplitude (*θ*_*a*_). Models with reduced or no random effects showed substantially poorer predictive performance, supporting the need to model between-person heterogeneity in RT adaptation. Accordingly, the model with random decay rate (*θ*_*b*_) and postadaptation RT (*θ*_*c*_) was selected for subsequent analyses.

#### Model Adequacy and Diagnostics

Building on the selected random-effects structure (random *θ*_*b*_ and *θ*_*c*_), adding responder-status moderation produced a small but consistent gain in out-of-sample predictive performance relative to the nonmoderated model (ELPD-LOO: −457.2 vs −459.7; SE≈26.6‐26.7). Although the absolute improvement was modest, it suggests that responder status explains some systematic variation in RT trajectories beyond subject-to-subject heterogeneity. LOO influence diagnostics were stable (99.7% of observations with Pareto *k*≤0.7), indicating that the model’s predictive evaluation was not driven by a small set of highly influential observations and that PSIS-LOO approximations were reliable. Markov chain Monte Carlo diagnostics further supported reliable posterior inference. Trace plots showed good mixing with no visible chain separation (Figure 1B), and convergence metrics (*R*≈1.00–1.01 with generally high ESS) indicated that posterior summaries are numerically stable and unlikely to reflect sampling pathologies. Finally, posterior predictive checks demonstrated that the model captures the key empirical pattern of interest, simulated trajectories reproducing the overall decay trend, and binned observed means closely tracked posterior predictive check medians while remaining largely within the 94% predictive intervals on the original RT scale (Figure 1C). Together, these checks suggest that the moderated model provides an adequate and generalizable description of RT dynamics in this sample, supporting the downstream interpretation of responder-nonresponder differences in adaptation parameters.

#### Responder Versus Nonresponder Differences in RT Adaptation

Responders exhibited markedly faster RT adaptation for the feeling item than nonresponders (Table 5). The responder-to-nonresponder ratio for the decay rate was substantially greater than 1 (median *θ*_*b*_ ratio=4.86, 95% CrI 1.44-14.31; *P* (increase)=.99), indicating a steeper decline (4.86 times faster adaptation) in RT over repeated EMA administrations. Responders also exhibited higher postadaptation RT level than nonresponders (median exp (*θ*_*c*_) ratio=1.25), indicating 1.25 times slower response after repeated administrations. However, group differences in the postadaptation RT level were more uncertain (95% CrI 0.95-1.58; *P* (increase)=.95), suggesting at most modest separation in late-phase baseline latency.

**Figure 2.**
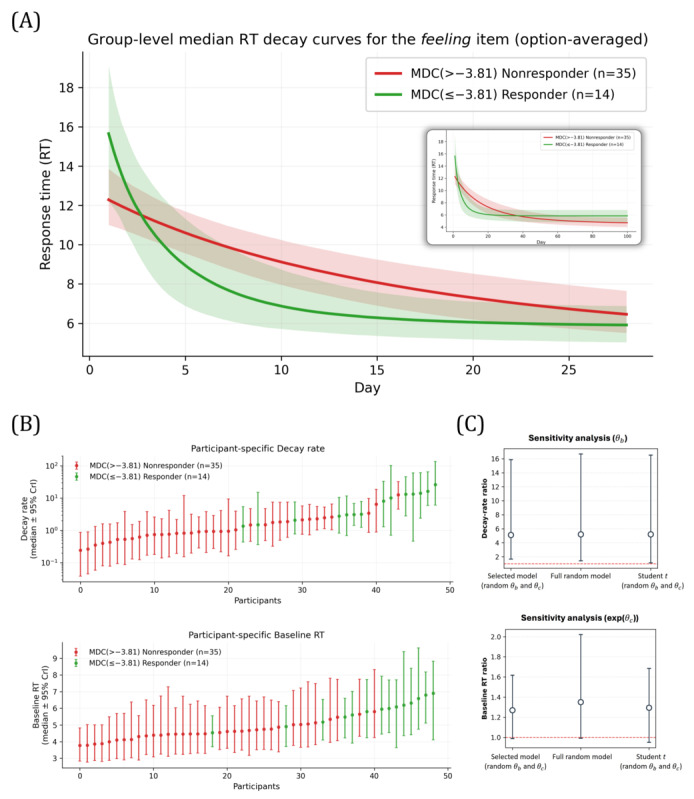
Responder-status (minimally detectable change [MDC]) moderation results for the feeling item. (A) Model-implied response time (RT) trajectories by group (posterior median with 95% credible interval [CrI]). The shaded region shows an illustrative extrapolation of the fitted trajectories up to 100 days. (B) Participant-level estimates for key parameters (*θ*_*b*_ and *θ*_*c*_) by group. (C) Sensitivity analysis of group-effect ratios across alternative model specifications.

Group-averaged trajectories were consistent with the posterior estimates, showing a steeper RT decline among responders (Figure 2A). Differences in the postadaptation RT level became more apparent when model-implied trajectories were extrapolated to 100 days beyond the observed study window (shaded region in Figure 2A). Subject-specific estimates supported the group-level findings (Figure 2B). Although posterior estimates varied within each group, such that some responders exhibited slower adaptation or lower baseline RT (and vice versa), individual trajectories generally followed the overall group trend. Consistent with this, rank plots of participant-level parameters showed responders tending to appear toward higher values of the decay rate and postadaptation RT level, indicating generally larger estimated values.

**Table 5.** Group moderation effects across EMA domains^a^.

Quantity and EMA^b^ item		Posterior median (IQR; width; 95% CrI^c^)	Posterior *P* (increase)
Decay rate (*θ*_*b*_) ratio			
Feeling		4.86 (3.524-7.609; 4.085; 1.438-14.31)	0.99
Appetite		2.22 (1.565-3.169; 1.604; 0.793-6.561)	0.93
Sleep quality		1.69 (1.186-2.373; 1.187; 0.608-4.707)	0.84
General evaluation		3.32 (1.828-6.229; 4.4; 0.593-19.26)	0.92
Average		3.11 (2.117-4.592; 2.475; 0.991-10.86)	0.97
Baseline RT (exp (*θ_c_*)) ratio			
Feeling		*1.25* (1.164-1.38; 0.2164; 0.953-1.581)	0.95
Appetite		1.04 (0.975-1.102; 0.1267; 0.862-1.247)	0.66
Sleep quality		0.96 (0.9008-1.025; 0.1244; 0.792-1.161)	0.33
General evaluation		1.09 (0.9891-1.2; 0.2108; 0.813-1.433)	0.73
Average		1.08 (1.015-1.154; 0.1392; 0.886-1.315)	0.80

a Ratios >1 indicate larger values in responder than nonresponder.

b EMA: ecological momentary assessment.

c CrI: credible interval.

#### Item-Specific Moderation Across EMA Domains

Additionally, moderation effects were examined across individual EMA items and an averaged RT outcome. Evidence for group separation was strongest for the feeling item, which showed the largest and most certain decay-rate difference (*θ*_*b*_ ratio=4.86, 95% CrI 1.44-14.31; *P*=.99). Other items and the averaged outcome showed smaller, less certain decay-rate ratios with CrIs generally overlapping 1. A similar pattern was shown for the postadaptation RT level, the feeling item again showed the largest (but still uncertain) group ratio (exp (*θc*) ratio=1.25, 95% CrI 0.95-1.58), whereas the remaining items showed minimal differences with intervals spanning 1.

#### Sensitivity Analyses

Group-effect conclusions for the feeling item were robust across plausible alternative specifications, including a full random-effects model and a Student *t* likelihood or alternative priors (Figure 2C). The responder-to-nonresponder decay-rate ratio remained consistently around 5 (~4.8), with 95% CrIs entirely above 1 across specifications, indicating a stable group difference in RT adaptation speed. By contrast, the postadaptation RT ratio remained modest (~1.3), and its CrIs overlapped 1 in all specifications, suggesting that late-phase RT differences are small and uncertain in this dataset.

## Discussion

### Summary of Results

This study provides preliminary evidence for the feasibility and potential efficacy of EMA-based self-monitoring in reducing psychological symptoms among older adults at elevated risk. Across 4 validated scales (CESD-R, PHQ-9, BAI, and GDS-15), participants showed statistically significant symptom reductions over the 4-week period, with medium-to-large effect sizes. These findings align with prior work suggesting that EMA-related benefits may be enhanced when used as an adjunct to treatment as usual, and they extend that evidence to a geriatric context. The high adherence to the EMA mobile app (>90%) further indicates that EMA-based self-monitoring is feasible and acceptable in older adults, including those with limited familiarity with mobile technologies, supporting its promise as a scalable adjunct to depression care in late life.

Correlational analyses also indicated that EMA-derived features can predict variation in symptom change, but with important limitations. Descriptive statistics of EMA scores and raw EMA RTs showed symptom-specific associations across measures, yet they were not significantly related to changes in the geriatric depression scale (ΔGDS-15), suggesting reduced sensitivity for tracking geriatric depressive symptom change using simple descriptives alone. In contrast, features derived from exponential-decay parameters fitted to standardized response times (Z-RT) showed consistent associations with depressive symptom change (ΔGDS-15 and ΔCESD-R). In particular, faster adaptation (higher decay rate), higher postadaptation RT level (asymptote/baseline), and better fit quality for the emotionally salient feeling item are associated with greater symptom improvement, suggesting that RT dynamics during EMA engagement may reflect clinically meaningful change during EMA-adjunctive care.

These patterns were further corroborated by the Bayesian multilevel modeling results. Compared with nonresponders, responders to EMA-adjunctive care exhibited markedly faster adaptation (higher decay rate) and modestly higher postadaptation RT levels. This suggests that potential responders show a distinct trajectory characterized by more rapid accommodation to repeated self-reflection, although the underlying mechanism cannot be determined from the current design (ie, whether EMA-adjunctive care enhances self-monitoring efficiency, or whether individuals with greater baseline capacity for such adaptation are more likely to benefit). Evidence for between-group differences in the postadaptation RT level was comparatively uncertain, and the direction (ie, slightly higher baseline RT in responders) may appear counterintuitive if faster responding is assumed to reflect greater efficiency. One plausible interpretation is that faster late-phase responding among nonresponders could reflect a tendency toward more superficial or less attentive responding rather than more efficient self-reflection, but this remains speculative and warrants direct validation. Additionally, sensitivity analyses across plausible alternative specifications yielded the same qualitative conclusions, supporting the robustness of the central group-difference inference. Collectively, these results highlight the potential clinical use of behavioral signatures embedded in EMA responding, particularly RT adaptation dynamics, for tracking depressive symptom improvement in older adults.

Interestingly, consistent with the correlational findings, group contrasts were strongest when the model was fitted to the feeling item among the 4 EMA domains examined. Moreover, although prior work suggests that averaging RT across multiple EMA items can increase measurement reliability [14,16], group differences in adaptive patterns were larger when using the feeling item alone. This finding suggests that EMA response dynamics may be item-specific rather than purely reflecting a global response-speed trait, raising the possibility that different symptom domains could exhibit distinct behavioral signatures in EMA trajectories.

### Clinical Implications

These findings suggest that EMA-adjunctive care may be both feasible and clinically beneficial for older adults at risk of depression. Digital mental health interventions (DMHIs) are often considered difficult to implement in late-life populations because limited familiarity with digital tools can reduce sustained engagement. In contrast, adherence in this study remained high (on average >90%), indicating strong real-world acceptability. The EMA protocol used here, brief daily reporting on four items, appeared manageable even for participants who might be expected to show reduced willingness or capacity for sustained participation due to depressive burden. To support sustained engagement in real-world deployment, the intended implementation can incorporate an explicit feedback loop, such as clinician-facing dashboards, periodic review during EMA engagements, or automated triggers that prompt timely outreach when a missing response is detected. Importantly, symptom reductions observed across validated scales indicate that EMA use in this context may confer benefits beyond feasibility, supporting EMA-adjunctive care as a promising component of geriatric depression management.

A second implication concerns the objective monitoring of symptom change. Remote psychiatric care still relies heavily on self-reported symptoms, ranging from retrospective standardized questionnaires to daily EMA ratings, both of which remain vulnerable to recall bias and other reporting distortions (eg, mood-congruent evaluation, self-reflective bias). EMA-derived RT metrics are not fully “passive” because they require active responding; however, they provide a comparatively objective behavioral signal that reflects underlying cognitive and psychomotor processes and is less directly shaped by deliberative self-presentation or response framing. The observed association between RT adaptation dynamics and clinically meaningful symptom improvement, therefore, supports RT trajectories as a complementary marker for tracking depressive symptom variation in naturalistic settings, potentially improving reliability when subjective reports are noisy or inconsistent.

Moreover, EMA-RT adaptation features may help make digital mental health interventions more actionable and personalized. As noted in the *Introduction* section, the effects of ecological momentary interventions are heterogeneous and depend on individual characteristics. Consequently, a practical barrier to scaling digital care is identifying early on who will benefit from a given protocol and who will require additional support. Our results suggest that RT adaptation patterns within an initial monitoring window (approximately 1 month) may provide an early indicator of EMA responsiveness. Such early stratification could guide stepped-care decisions (eg, prompting intensified clinician contact, alternative interventions, or safety monitoring for likely non-responders) while allowing responders to continue with lower-intensity, self-guided support, thereby supporting more efficient allocation of constrained clinical resources and optimizing just-in-time adaptive intervention approaches. We also note that this early stratification window may be shortened through systematic investigation of the optimal decision period. Specifically, building on the observed faster adaptation among responders, future work could quantify within-person day-to-day EMA-RT gradients (or slopes over rolling windows) and evaluate candidate “decision days” (eg, 7, 10, 14, or 21 days) to determine an earlier, reliable point for stepped-care decisions.

Finally, the item-specific links observed between EMA domains and symptom changes suggest a pathway to broaden EMA monitoring beyond depression. Because psychiatric symptoms frequently co-occur, identifying domain-specific behavioral signatures (eg, the feeling-depression linkage observed here) may enable EMA to track multiple symptom dimensions simultaneously, supporting more nuanced, individualized monitoring and intervention planning.

### Limitations

First, regarding the observed effectiveness of the EMA-based self-monitoring, although significant reductions in depressive and anxiety symptoms were detected across multiple validated scales, the absence of a control group precludes definitive causal attribution. Participants were not randomized, and potential concurrent treatments, such as case management, pharmacotherapy, or psychotherapy, were not systematically recorded or controlled, introducing possible confounding influences. Additionally, postintervention assessments were conducted only once at the 4-week mark without follow-up measurements to evaluate the durability of treatment effects. As such, the long-term efficacy and sustainability of EMA-based self-monitoring in older adults with depression remain undetermined.

While several EMA-derived features were shown to be associated with symptom variation, some unexpected patterns emerged in the relationship between self-reported EMA scores and symptom change. Specifically, positive correlations were observed between mood-related EMA scores and symptom change in both depression and anxiety domains. These findings suggest that higher EMA ratings during the intervention period were paradoxically linked to less clinical improvement. One possible interpretation is that individuals with persistent depressive symptoms may have reduced self-reflective precision, leading to inflated or undifferentiated positive reports. Alternatively, healthier participants may have demonstrated more nuanced and accurate self-monitoring. However, this hypothesis requires further empirical validation.

Additionally, given the modest sample size (n<50), the correlational analyses were likely underpowered to detect small associations between symptom-change scores and EMA-derived features; thus, null or weak correlations should be interpreted cautiously. Larger cohorts will be needed to more precisely estimate small effects and confirm the robustness and generalizability of the observed associations, particularly those involving the exponential-decay parameters.

Finally, in examining dynamic EMA response trajectories, the most distinctive group-level differences between responders and non-responders emerged in the fitted parameters of the feeling item, whereas appetite, sleep quality, and general evaluation items yielded less pronounced contrasts. This discrepancy may reflect the unique psychological salience of the feeling item, which directly targets the immediate emotional state, in contrast to the more somatically anchored or abstract content of the other items. Alternatively, the fixed order of EMA item presentation, wherein the feeling item was always administered first, may have contributed to its stronger predictive utility. It is plausible that the first item elicits more cognitive effort or reflects the initiation speed of self-reflective responses, thus capturing more variance in related cognitive functioning. This potential order effect should be systematically examined in future studies to better delineate what EMA-based response latencies truly index in ecological settings.

### Conclusions

This study demonstrates the feasibility of using EMA-based self-monitoring as both an adjunctive care and a behavioral phenotyping tool in older adults with a high risk of depression.

The implications for digital health care are manifold. First, the integration of response-time dynamics into mobile health platforms offers a nonobtrusive means of tracking cognitive and emotional engagement, even in populations with limited digital literacy. The method’s compatibility with brief, low-frequency EMA schedules (eg, daily reports, about a month) makes it especially suitable for aging individuals, who may be less tolerant of intensive digital protocols. Second, the use of within-subject standardization and curve-fitting models supports individualized tracking, allowing for the detection of meaningful intra-individual changes. This has significant implications for the early identification of symptom relapse, monitoring of treatment responsiveness, and tailored interventions based on dynamic behavioral signatures.

In sum, the introduced EMA-based modeling offers a novel, sensitive, and pragmatic approach for advancing mental health care in aging populations. Future work should aim to validate these findings in randomized controlled settings, investigate the mechanistic underpinnings of RT adaptations, and explore broader applications in digital psychiatry and geriatric care.

## Supplementary material

10.2196/83891 Multimedia Appendix 1 Methodological details.

10.2196/83891 Multimedia Appendix 2 Correlational analysis table.

## Abbreviations

BAI: Beck Anxiety Inventory
CESD-R: Center for Epidemiologic Studies Depression Scale–Revised
CrI: credible interval
*DSM-5*: *Diagnostic and Statistical Manual of Mental Disorders, Fifth Edition*
ELPD-LOO: expected log pointwise predictive density leave-one-out cross-validation
EMA: ecological momentary assessment
GDS-15: 15-item Geriatric Depression Scale
MDC: minimal detectable change
MDD: major depressive disorder
PHQ-9: 9-item Patient Health Questionnaire
PSIS-LOO: Pareto-smoothed importance sampling leave-one-out cross-validation
RT: response time
