# Supplementary material for: Response Time Dynamics From Noncognitive Ordinal Ecological Momentary Assessment as a Proxy for Symptom Change in Geriatric Depression: Longitudinal Observational Study
Source: JMIR Aging. 2026 May 8;9:e83891. doi: 10.2196/83891 (PMC13154371; doi:10.2196/83891)
Supplement: Multimedia Appendix 1 [file aging-v9-e83891-s001.docx]

Supplementary Materials **List of contents**

1. Defining outlier thresholds for non-cognitive ordinal EMA response time
2. Technical details for fitting subject-wise exponential-decay model
3. Model specifications for bayesin multilevel modeling

**Supplementary tables**

Table S1. Predefined lower and upper boundaries of response time utilized for analysis

Table S2. Prior distribution configuration used in multilevel exponential decay model

**Supplementary figures**

Figure S1. Histograms of response time for each EMA items

**I. Defining Outlier Thresholds for Non-Cognitive Ordinal EMA Response Time**

Outlier boundaries adequate for Ecological momentary assessment(EMA)-based response time analysis were determined considering population characteristics, task difficulties and dataset distribution. Outlier of response times in traditional cognitive tasks are determined by unusual observation that lies outside the within-subject range. (normally, Mean ±1 SD). However, such procedure requires numerous trials for each sujects (typically more than 100 trials), thus is not applicable/less practical in EMA settings.

We instead took data-driven, populational approach to define adequate RT boundaries. **Figure S1** indicates histograms of response time obtained from all participants for each EMA items (*feeling*, *appetite*, *sleep quality*, *general evaluation*). As visualized, the distributions of response time are heavily right skewed. Applying traditional Inter Quantile Range(IQR) rule, where lower bound is defined as Q1 - 1$\times$IQR and upper bound is defined as Q3 + 1$\times$IQR, the lower bound values reach negative values including even the responses near zero that may have resulted from careless responding. Also, upper boundary precludes much of the observations from several subjects that shows slow average response times, which may be of particular importance since the main focus is of old age with depression characterstic of psychomotor slowing.

**Figure S1.** Histogram of response time for each EMA items (feeling, appetite, sleep quality, general well-being)


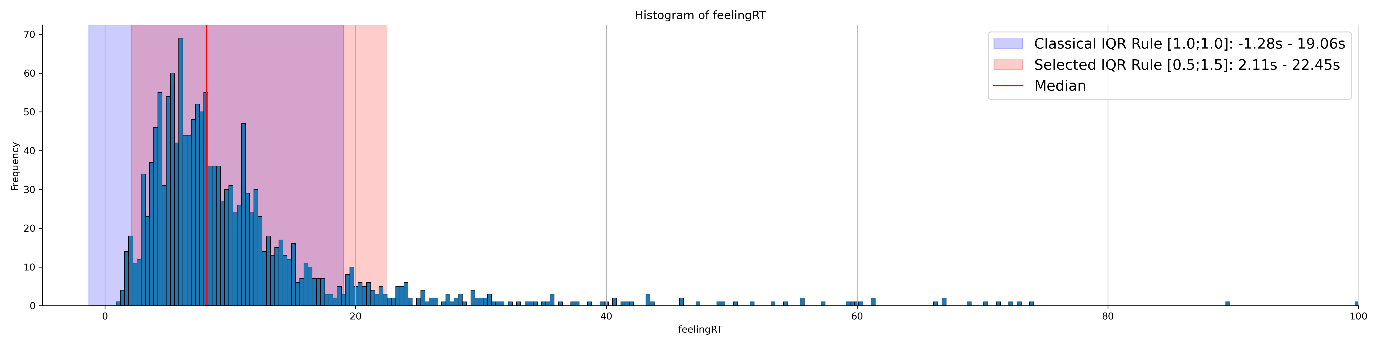


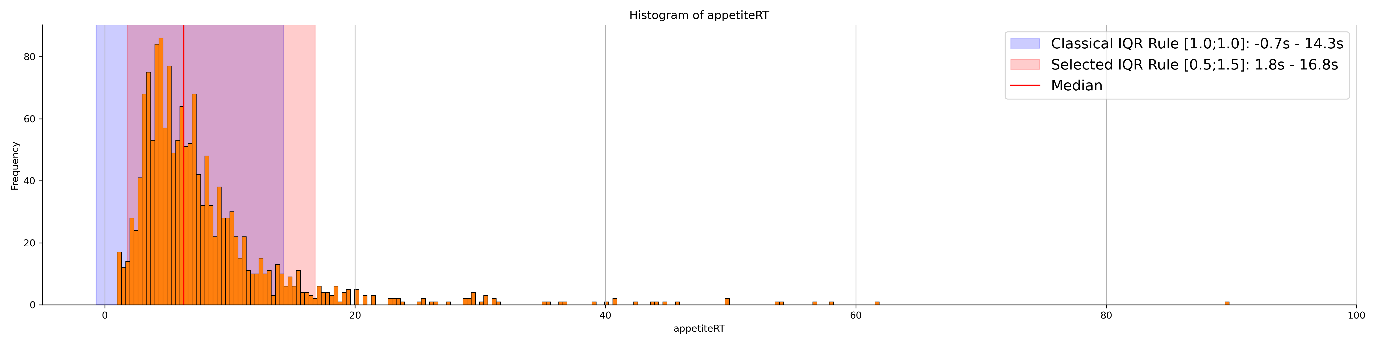


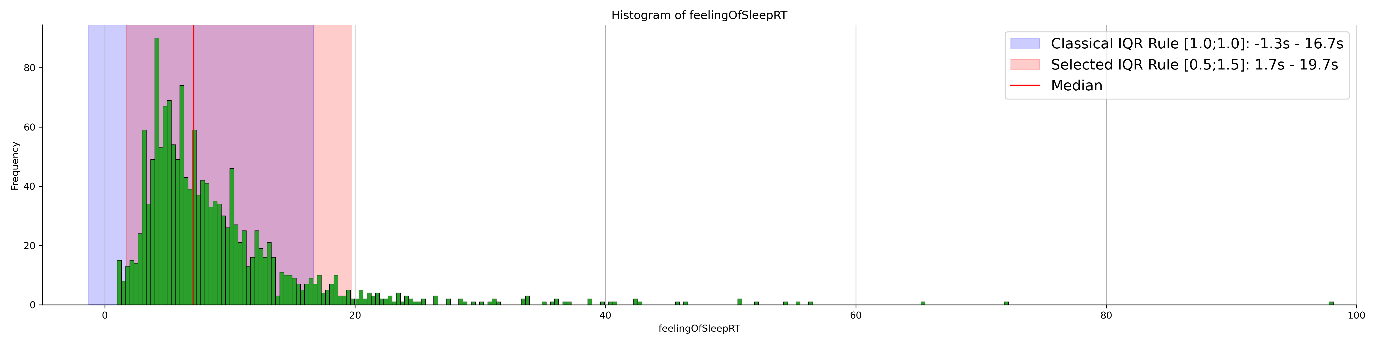


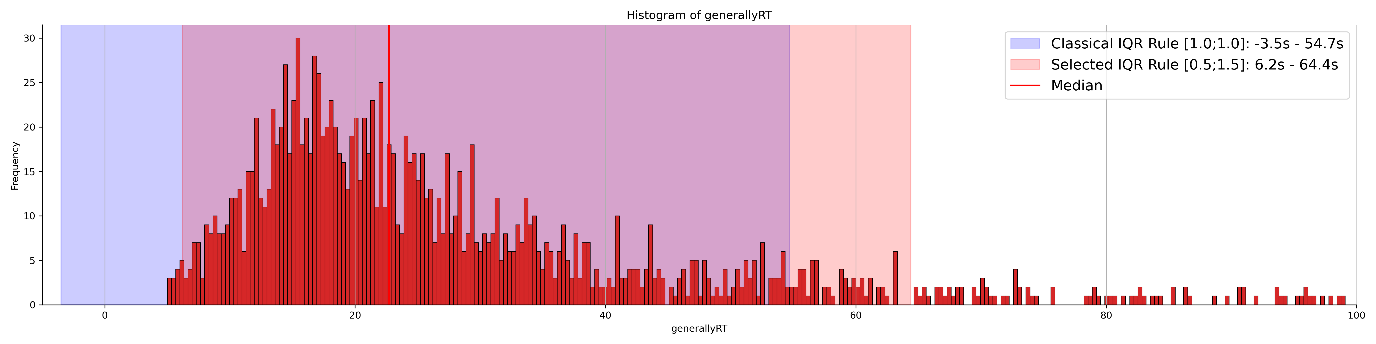


Thus, we modified the IQR rule of thumb that is suitable for our dataset. A lower bound was defined as Q1 – 0.5$\times$IQR and upper bound was defined as Q3 + 1.5$\times$IQR. With the predefined boundaries, subject that had the minimum trials were 13days which roughly matches to w weeks of EMA usage. Boundaries for each EMA items are listed at **Table S1**.

**Table S1.** Predefined lower and upper boundaries of response time utilized for analysis

|  | Lower bound (s) | Upper bound (s) |
| --- | --- | --- |
| *feeling* | 2.11 | 22.45 |
| *appetite* | 1.8 | 16.8 |
| *sleep quailty* | 1.7 | 19.7 |
| *general evaluation* | 6.2 | 64.4 |

**II. Technical Details for Fitting the Subject-wise Exponential-Decay Model**

We modeled each participant $\times$ EMA response time series with a simple exponential-decay curve $f(x)=a\cdot e^{-bx}+c$, where $a$ is how much slower the early trials are above the stable level, $b$ is how fast RT improves, and $c$ is the long-run RT. We fit this curve with SciPy’s curve_fit using bounds to keep parameters in sensible ranges and started the trial index at $x=1$. For raw response times, we used broad, data-driven starting values and wide bounds so the fit can handle different baseline speeds and devices ($p0=\left[ max\left( y \right),0.1,min\left( y \right) \right], a\in\left[ 0,100 \right], b\in[0,\infty], c\in[min(y),max(y)]$). For within-person Z-transformed response times, we used tighter starts and bounds because the series are already centered and on a comparable scale($p0=\left[ max\left( y \right),1.0,min\left( y \right) \right], a\in[0,4],b\in[0,3],c\in[min(y)-1,max(y)+1]$). We summarized fit quality with $R^{2}$ and set a generous iteration limit (maxfev = 10,000) to avoid early stopping.

**III. Model Specifications for Bayesian Multilevel Exponential Decay model**

1. *Likelihood and Observation Model*

The reaction time $RT_{n}$ for each observation $n=1,\ldots,N_{obs}$ is assumed to follow a Log-Normal distribution. This ensures that predicted RTs remain positive and accounts for the typical right-skewed nature of RT data.

$$RT_{n}\sim LogNormal\left( \eta_{n},\sigma_{log} \right)$$

Equivalently, on the logarithmic scale:

$$\log\left( RT_{n} \right)\sim Normal\left( \eta_{n},\sigma_{log}^{2} \right)$$

Where $\eta_{n}$ is the location parameter (mean of the log-transformed RT) and $\sigma_{log}$ is the scale parameter representing residual observation noise.

2. *The Mean Function (Latent Process)*

The mean function $\eta_{n}$ follows an exponential decay process to model the change in RT over time. The structured form is defined as:

$$\eta_{n}= \theta_{a}^{\left( i \right)}\cdot\exp\left( -\theta_{b}^{\left( i \right)}\cdot t \right)+\theta_{c}^{\left( i,r \right)}$$

Let $i$ denote the subject index, $r$ the response option(1,…,7 on a Likert scale), and $t\in\left[ 0,1 \right]$ the normalized index. The parameters are defined as:

$\theta_{a}$(Amplitude): The initial elevation above the asymptote at $t=0$.

$\theta_{b}$(Decay Rate): The speed at which the RT approaches the baseline.

$\theta_{c}^{\left( i,r \right)}$(Asymptote/Baseline): The subject-specific and response-specific baseline on the log-RT scale.

3. *Hierarchical Structure and Moderation*

To facilitate robust estimation and account for individual differences, we use a non-centered parameterization. This improves Hamiltonian Monte Carlo (HMC) sampling efficiency by decoupling individual-level effects from group-level priors.

A. Amplitude ($\theta_{a}$) and Decay Rate ($\theta_{b}$)

We use a log-link function for $\theta_{a}$ and $\theta_{b}$ to ensure these parameters remain strictly positive. Both are moderated by a covariate $g^{\left( i \right)}$ (e.g., MDC threshold group):

$$log\theta_{a}^{\left( i \right)}={\beta0}_{a}+{\beta1}_{a}\cdot g^{\left( i \right)}+\sigma_{a}\cdot z_{a}^{\left( i \right)},z_{a}^{\left( i \right)}\sim Normal\left( 0,1 \right)$$

$$log\theta_{b}^{\left( i \right)}={\beta0}_{b}+{\beta1}_{b}\cdot g^{\left( i \right)}+\sigma_{b}\cdot z_{b}^{\left( i \right)},z_{b}^{\left( i \right)}\sim Normal\left( 0,1 \right)$$

B. Asymptote Decomposition ($\theta_{c}$)

The baseline $\theta_{c}^{\left( i,r \right)}$ is modeled directly on the log-RT scale. It is decomposed into fixed effects (group moderation and response option) and random effects(suject-level and cell-level residuals):

$$\theta_{c}^{\left( i,r \right)}=\underset{\text{Group-Moderated Baseline}}{\underbrace{\left( {\beta0}_{c}+{\beta1}_{c}\cdot g^{\left( i \right)} \right)}}+\underset{\text{Subject Intercept}}{\underbrace{u_{c}^{\left( i \right)}}}+\underset{\text{Option Main Effect}}{\underbrace{\alpha_{c}^{\left( r \right)}}}+\underset{\text{Cell Residual}}{\underbrace{\epsilon_{c}^{\left( i,r \right)}}}$$

Where:

${\beta0}_{c}$: The global basline intercept.

$u_{c}^{\left( i \right)}$: The subject-level random intercept, where $u_{c}^{\left( i \right)}\sim Normal\left( 0,\sigma_{subj}^{2} \right)$.

$\alpha_{c}^{\left( r \right)}$: The fixed effect of response option $r$ (Likert 1-7), capturing inherent processing differences between choices.

$\epsilon_{c}^{\left( i,r \right)}$: The cell-specific residaul (subject $\times$ response option interaction), where $\epsilon_{c}^{\left( i,r \right)}\sim Normal\left( 0,\sigma_{cell}^{2} \right)$.

This decomposition aligns with frameworks advocating for the within-person standardization of RTs prior to downstream feature extraction. By explicitly modeling these components, the model accounts for response-option biases in observed latency while simultaneously partitioning subject-specific baseline variance.

To ensure the model is identifiable, the option main effects ($\alpha_{c}$) are subject to a sum-to-zero constraint:

$$\sum_{r=1}^{R} \alpha_{c}^{\left( r \right)}=0$$

Under this constraint, $u_{c}^{\left( i \right)}$ identifies the global average baseline across all subjects and Likert options, while $\alpha_{c}^{\left( r \right)}$ identifies the relative processing cost of selecting a specific response option.

4. *Prior Distributions*

Priors are chosen to be weakly informative, providing regularization while allowing the data to drive the posterior distributions.

**Table S2.** Prior distribution configuration used in multilevel exponential decay model

| Parameter | Notation | Prior Distribution | Rationale & Identification |
| --- | --- | --- | --- |
| Population Intercepts | ${\beta0}_{a,b,c}$ | $\text{Normal}\left( \mu_{emp},3^{2} \right)$ | Centered on empirical log-quantiles ($p_{10}and p_{90}$) to facilitate stable convergence. |
| Moderation Slopes | ${\beta1}_{a,b,c}$ | $\text{Normal}\left( 0,3^{2} \right)$ | Weakly informative; assumes no baseline group differences (null effect). |
| Response Option Effects | $\alpha_{c}^{\left( r \right)}$ | $\text{Normal}\left( 0,1^{2} \right)$ | Subject to a sum-to-zero constraint ($\sum\alpha_{c}=0$) to ensure global intercept identifiability. |
| Group-Level Scale | $\sigma_{a},\sigma_{b}$ | $\text{Half-Normal}\left( 0,1.5 \right)$ | Regularizes interpersonal variability in amplitude and decay rate. |
| Random Effects Scale | $\sigma_{\text{subj}},\sigma_{\text{cell}}$ | $\text{Half-Normal}\left( 0,1.5 \right)$ | Constrains subject-specific intercepts and cell-level interaction residuals. |
| Residual Noise | $\sigma_{\text{log}}$ | $\text{Half-Normal}\left( 0,1 \right)$ | Standard weakly informative prior for residual variance on the log-RT scale. |

5. *Sampling Configurations*

Bayesian parameter estimation was carried out using Hamiltonian Monte Carlo with the No-U-Turn Sampler (NUTS) as implemented in PyMC. Given the hierarchical structure and correlated posteriors typically induced by multilevel exponential-decay models, we used a conservative sampling configuration to promote stable exploration of the posterior. Specifically, we ran 4 independent MCMC chains in parallel (4 cores), each consisting of 1,000 tuning (warm-up) iterations followed by 1,000 retained posterior draws, yielding 4,000 post–warm-up samples in total. Chains were initialized with the ‘adapt_diag’ strategy, and the NUTS target acceptance probability was set to 0.95 to mitigate divergent transitions and improve numerical stability.
